# Secured and Privacy-Preserving Multi-Authority Access Control System for Cloud-Based Healthcare Data Sharing

**DOI:** 10.3390/s23052617

**Published:** 2023-02-27

**Authors:** Reetu Gupta, Priyesh Kanungo, Nirmal Dagdee, Golla Madhu, Kshira Sagar Sahoo, N. Z. Jhanjhi, Mehedi Masud, Nabil Sharaf Almalki, Mohammed A. AlZain

**Affiliations:** 1 School of Computer Science and Information Technology, Devi Ahilya Vishwavidyalaya, Indore 452001, India; 2 SKITM College, Indore 452020, India; 3 Department of Information Technology, VNR Vignana Jyothi Institute of Engineering and Technology, Hyderabad 500090, India; 4 Department of CSE, SRM University, Amaravati 522240, India; 5 School of Computer Science, SCS Taylors University, Subang Jaya 47500, Malaysia; 6 Department of Computer Science, College of Computers and Information Technology, Taif University, Taif 21944, Saudi Arabia; 7 Department of Special Education, College of Education, King Saud University, Riyadh 145111, Saudi Arabia; 8 Department of Information Technology, College of Computers and Information Technology, Taif University, Taif 21944, Saudi Arabia

**Keywords:** electronic health records, access control, cloud storage, attribute-based encryption, multiple authorities, privacy preservation

## Abstract

With continuous advancements in Internet technology and the increased use of cryptographic techniques, the cloud has become the obvious choice for data sharing. Generally, the data are outsourced to cloud storage servers in encrypted form. Access control methods can be used on encrypted outsourced data to facilitate and regulate access. Multi-authority attribute-based encryption is a propitious technique to control who can access encrypted data in inter-domain applications such as sharing data between organizations, sharing data in healthcare, etc. The data owner may require the flexibility to share the data with known and unknown users. The known or closed-domain users may be internal employees of the organization, and unknown or open-domain users may be outside agencies, third-party users, etc. In the case of closed-domain users, the data owner becomes the key issuing authority, and in the case of open-domain users, various established attribute authorities perform the task of key issuance. Privacy preservation is also a crucial requirement in cloud-based data-sharing systems. This work proposes the SP-MAACS scheme, a secure and privacy-preserving multi-authority access control system for cloud-based healthcare data sharing. Both open and closed domain users are considered, and policy privacy is ensured by only disclosing the names of policy attributes. The values of the attributes are kept hidden. Characteristic comparison with similar existing schemes shows that our scheme simultaneously provides features such as multi-authority setting, expressive and flexible access policy structure, privacy preservation, and scalability. The performance analysis carried out by us shows that the decryption cost is reasonable enough. Furthermore, the scheme is demonstrated to be adaptively secure under the standard model.

## 1. Introduction

The modern health industry is adopting Internet of Things (IoT) technology for providing advanced healthcare services [1]. A wide range of IoT devices and applications are designed for healthcare needs, e.g., sensors, remote healthcare monitoring applications, telemedicine consultation applications, etc. Healthcare organizations can collect, record, and monitor patient data regularly, providing them with adequate treatment in every situation. Patients can be treated well in emergencies by making use of their electronic health records (EHRs). For example, let us assume that a patient is suffering from chronic heart disease and they use body sensors to record their blood pressure. As patient monitoring services make their EHR available online, their doctors can treat them efficiently. In addition, in some unfavorable conditions when they may need medical attention, any treating doctor can access their data to make the right diagnosis. The availability of their EHRs can save their life.

Patients can maintain their EHR data on cloud storage servers and receive various advantages such as 24/7 access, easy management, and tracking and sharing of the EHR data for treatment as well as beneficiary purposes. However, cloud-based data outsourcing is not trusted because the data owners lose physical control of their data. Performing data encryption before uploading to the cloud is one solution to the above problem. This limits accessibility for legitimate users, as they will need decryption keys for accessing the data. Data owners can only issue the keys to known users. Other legitimate users cannot access the encrypted data in this case. Therefore, there is a need for a secure data-sharing system allowing the data owner to provide access control to data in a fine-grained manner. The system should have proper attributes and key management facilities to serve all legitimate users. To accomplish this outsourcing paradigm, the cloud-based EHR approach is motivated by the concepts of “Availability, Scalability, Cost efficiency, and Convenience” [2]. Dissemination of patient data through cloud-based EHRs is advantageous, but it must be performed with such care that the privacy of patients is protected.

Various cryptographic access control models have been defined to provide data security and control unauthorized data access. For this need, attribute-based encryption, also known as ABE [3], seems to be a suitable option. It allows for fine-grained and flexible access control. One type of ABE called ciphertext-policy attribute-based encryption [4] or CP-ABE is a technique in which the user’s secret key is generated for their attributes and an access policy is coupled with ciphertext. Access policies are typically expressed by threshold gates or AND-OR gates over the attributes. Users receive their attribute keys in terms of secret keys issued by the attribute authorities. CP-ABE is a kind of public key encryption (PKE) technique. It provides access control for a large group of users because it allows decryption only when the user carries the attribute set that matches the policy. Credentials or attributes are issued to the users by various attribute authorities, which may be from single or multiple domains. Several ABE techniques for cloud-based data sharing are given [5,6,7,8]. However, these schemes consider a single domain authority that manages all the attributes. In practical scenarios, users from multiple domains may request a download of the shared data and different administrating authorities may issue the attributes. Additionally, normal cipher-policy ABE schemes [4,9] store the access policy with outsourced encrypted data, but in plain text. This may result in the disclosure of sensitive information regarding the encrypted data. Let us understand cloud-based data sharing with the help of a healthcare use case.

### 1.1. Use-Case

**Figure 1:**
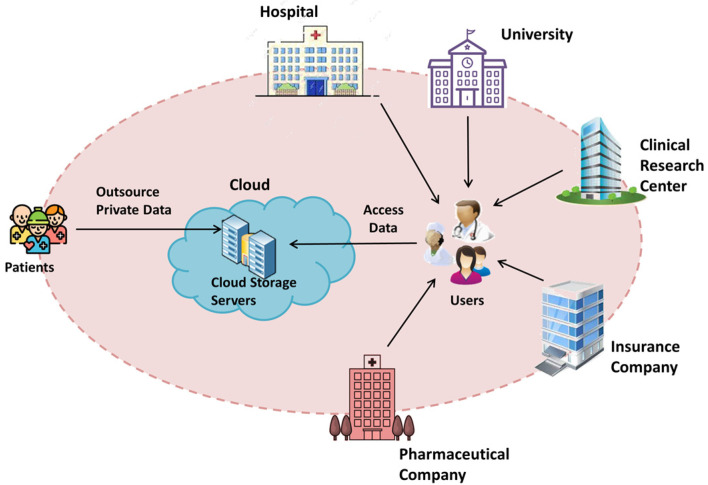
Cloud-based healthcare data sharing.

In the healthcare system, various users such as doctors, nurses, research scholars, insurance agents, etc., may need to access the data. In this case, various authorities, e.g., hospitals, clinical research centers, insurance companies, and pharmaceutical companies, may issue various attribute keys to the concerned users (as shown in Figure 1). The shared EHRs of the patients may be protected by instituting access policies, such as any user who possesses the “Research Assistant” attribute in the domain of renowned “Research Organization” or “Insurance agent” attribute in the domain of “Insurance Company” from where the patient is receiving services being permitted to access the data. Therefore, in healthcare industries, where the user fraternity is a large group, multi-authority CP-ABE schemes are a suitable option for facilitating efficient and secure EHR sharing. All the above-stated users in the healthcare system come from the open domain, i.e., they are not known to the data owner.

To control access to shared data, attributes issued by various open-domain attribute authorities are used in access policies. Another important issue that must be considered in EHR data sharing is to give access to closed-domain users, i.e., the users known to the data owner, e.g., family friends, relatives, etc. Flexibility in handling the access requirements of both closed- and open-domain users makes the sharing of health data more practical and effective [10]. In emergencies, family friends or relatives may access the data. The privacy of the patient is also crucial in this kind of data sharing. In the above instance, if a patient outsources their medical data with policy “[(Profession = “Doctor” and Specialization = “Cardiology” and Affiliation = “AIIMS”) or (Profession = “Doctor” and Specialization = “Cardiology” and Research Associate = “University Hospital”) or (Relation = “Friend”)]”, then everyone including adversaries and the cloud service provider (CSP) can look at the policy formulation and figure out that the shared data are of a patient who is suffering from heart disease. This results in privacy leakage even though the ciphertext of EHR data is protected well. Therefore, it is essential to keep the access policy a secret to protect sensitive data.

### 1.2. Related Works

Numerous researchers have discussed data outsourcing in the cloud environment. Attribute-based encryption introduced by Sahai-Waters [11] is considered as most prominent scheme to institute access control for encrypted data. Although ABE and similar systems [4,9,11,12] employ one-to-many encryption concepts, there are some concerns with these techniques. The single authority managing the key issuance process for all the eligible users may decrypt every ciphertext by using issued secret keys. This is called the key escrow problem. Another problem in these schemes is low system performance due to over-reliance on a single authority for handling all of the system’s keys. This motivated the concept of establishing multiple authorities for key management tasks in a distributed manner. Several multi-authority ABE schemes and schemes supporting policy privacy have been presented in the literature.

#### 1.2.1. Multi-Authority ABE Schemes

Multi-authority ABE was first discussed in the work [13], where only one CA, also known as the central authority, and numerous AAs, also known as attribute authorities, controlled key management. The CA and AAs were responsible for issuing keys for identity and keys for attributes, respectively. The use of the global identifier GID prevented user collusion problems. However, the CA was capable of decrypting any ciphertext. Chase and Chow [14] improved their scheme (CC-MA-ABE) by removing the CA and introducing an anonymous key distribution mechanism with the help of pseudo-random functions. Both schemes in [13,14] had the limitation that they supported an AND policy architecture only. Liu et al. [15] presented the MACP-ABE scheme, a fully secure scheme in the standard model where multiple central and attribute authorities collaborate to work together. The central authorities are responsible for issuing keys related to the user’s identity, and the attribute authorities control the issuance of the attribute-related keys. Lewko and Waters [16] designed a decentralized CP-ABE and demonstrated security under the random oracle model. The linear secret sharing scheme, also known as LSSS, was used for specifying the access policy. At setup and key generation, no coordination between authorities was necessary, nor was there a central authority. Li et al. [17] proposed a scheme where the user’s community can be divided into public and personal domains (PUD and PSD, respectively) depending upon their role in the system. Their scheme was based on scheme [14] and policy was specified using a conjunctive normal form (CNF) structure. Ibraimi et al. [18] suggested a scheme for patient health record sharing in a multi-authority (two authorities) setting. They introduced social domains and professional domains for different authorized users. Several pieces were proposed to cater to different issues regarding MA-ABE. Ruj et al. [19] addressed the revocation function in a multi-authority setting. However, their method had heavy communication overhead and key update computation overhead. The authors of [20] proposed cipher policy ABE schemes for supporting multi-authority scenarios and handling user revocation features. To improve efficiency, the decryption process was outsourced. AA became a bottleneck in the scheme, as it had to calculate update keys for each unrevoked user. The scheme presented in [21] improved the CP-ABE scheme for secure PHR sharing [22] for the multi-authority scenario. The authors also defined public and private domains for their PHR-sharing scheme. Li et al. [23] introduced an access control scheme for cloud storage supporting decryption outsourcing. The scheme was also a multi-authority scheme and was evidenced to be adaptive and secure. In order to eliminate key escrow and minimize computation and communication costs, Hu et al. [24] provided an MA-ABE scheme that resisted key escrow and had a ciphertext of constant size. Furthermore, in the MA-KPABE scheme presented in [25], verifiability of partial decryption ciphertext (PDC) and delegation features were added. Ma et al. [26] presented two decentralized CP-ABE techniques for the standard model. The CAs and AAs operated independently of one another. The first technique was constructed using a group of composite orders. The second technique produced ciphertexts of constant size in the groups of prime order. The first technique worked for any monotonic structure, while the second worked for AND-gate policies.

#### 1.2.2. Policy Preservation in Attribute-Based Encryption

Because encrypted data on cloud storage servers remained in plaintext form when outsourced, access policies were shared with different users in all of the above schemes. This may cause potential exposure of sensitive information about the data owner as well as the consumers of the data. Several works [27,28] introduced CP-ABE schemes with partially hidden access control policies to protect the disclosure of this sensitive information. The access policies in these schemes divide attributes into two parts, i.e., attribute name and attribute value. In a partially hidden access policy, the attribute value that reveals sensitive information is made hidden, e.g., ([(Profession = “*” and Specialization = “*” and Affiliation = “*”) or (Profession = “*” and Specialization = “*” and Research Associate = “*”) or (Relation = “*”))). Li et al. [29] used the anonymous key issuing protocol used in CC-MA-ABE [14] and proposed an accountable multi-authority CP-ABE. Han et al. [30] developed a decentralized CP-ABE (PPDCP-ABE) scheme to eliminate dependency on and trust in central authorities while maintaining the privacy of users. In their scheme, multiple authorities may operate autonomously. The Pedersen commitment protocol [31] and zero-knowledge-proof protocol [32] were used to protect the attributes’ privacy. In [32], a policy-hiding CP-ABE scheme was proposed to improve decryption efficiency. The authors pioneered the “match-then-decrypt” method, in which ciphertext components were routed to the decryption test. Without performing the actual decryption, it checked the satisfiability of the hidden attributes policy for the attribute private key. Chen et al. [33] designed a privacy-preserving decentralized CP-ABE where the secret key was taken out with privacy. Their proposal required no central AA or multi-authority collaboration. They used the scheme [31] proposed by Pederson and oblivious attribute certificates [34]. The users receive secret keys for legitimate identity attributes, but AAs cannot find any useful information. Zhong et al. [35] proposed an access control scheme with a hidden policy on multi-authority architecture. To hide policy, attributes were obfuscated using a one-way anonymous key agreement protocol. Yang et al. [36] proposed a method for controlling Big Data access. Instead of hiding the attribute values, they hid the whole attribute for privacy purposes. They utilized an attribute bloom filter, which detects an attribute and its precise location in the access policy. Ying et al. [37] presented a lightweight policy-preserving CP-ABE scheme for EHR sharing on the cloud. The access policy was fully hidden by use of the attribute cuckoo filter (ACF). PASH, proposed in [38], was designed to provide access control for smart health. The CP-ABE scheme supported a large universe and used partially hidden policies. It also handled decryption tests efficiently and provided full security. Yan et al. [39] introduced a multi-authority ABE with privacy preservation and dynamic policy updating. This scheme was suggested for a multi-authority scenario, but it could not prevent malicious users from sharing their private keys. Belguith et al. [40] proposed PHOABE for cloud-assisted IoT, where multiple authorities were considered and a fully hidden policy was maintained by obfuscating the attributes. Their scheme was based on the scheme in [16]. It introduced a semi-trusted cloud server for outsourcing the heavy decryption process. This minimized computation overhead on resource-constrained devices. Zhang et al. [41] developed a hidden ciphertext-policy scheme for a large universe and proposed an efficient decryption procedure. Chinnasamy et al. [42] proposed a policy-hidden CP-ABE scheme for providing access control in an IoT environment. The SHA1 hashing algorithm was used for policy anonymization. Research works in [43,44] presented challenges related to cloud storage resources and applications in IoT. Najafi et al. [45] introduced a system with attribute privacy and search capabilities over encrypted data. In order to keep medical records safe and accessible, they created a storage and retrieval system. The approach was safe against keyword guess attacks in the standard model.

Analysis of the aforementioned schemes elucidated some comparative notes and observations. The following is a brief outline of issues with prominent MA-ABE schemes.

(1) Several attribute-based access control schemes [13,14,15,16,17,18,19,20,21,22,23,26] in the multi-authority domain have been proposed, but these methods lack the policy preservation aspect;
(2) There are schemes supporting privacy preservation approaches [27,28,29,30,32,33,35,36,37,38,39,40,41,46], but some of them are single-authority [27,38] and some of them use fully hidden access policies [35,40], which are more rigid in nature;
(3) The security of most of the multi-authority schemes was validated in weaker security models, i.e., selective security [35,39], where adversaries need to declare a challenged access policy structure before obtaining the public parameters. There is a requirement for higher security in the above scenario.

### 1.3. Our Contributions

To resolve the aforementioned problems, SP-MAACS, a secure and privacy-preserving multi-authority access control system for cloud-based data sharing is proposed. Our SP-MAACS is a secure MA-CP-ABE scheme with a partial policy hiding feature. Two components make up an attribute: the name and the value. Concrete attribute values are used in the access policy. They are encoded in the ciphertext components. The access policy in plaintext is also saved with the encrypted data. It includes attribute names, not values. In our scheme, we used the access policy in DNF form, also called “alternative routes to authorization” [47]. A set of satisfiable sub-policies can be derived from the main policy. For example, a policy for the data D represented as an arbitrary logical formula such as [a1 ∧ (a2 ∨ a3)] can be written as [(a1 ∧ a2) ∨ (a1 ∧ a3)]. Here the set of sub-policies is [(a1 ∧ a2), (a1 ∧ a3)]. Generally, when a data owner decides on an access policy, he starts framing it using a combination of alternatives. This standard method of specifying access policy corresponds to the DNF structure employed by our approach. The following is a concise summary of the primary features of our system:

→ The proposed scheme incorporates the important aspect of privacy preservation in a multi-authority setting. Along with this added-on feature of privacy preservation, our multi-authority access control scheme also achieves better decryption efficiency;
→ The scheme is designed to support open- and closed-domain users and allows for employing fine-grained access control. The access policy formulated using DNF makes the policy specification more flexible and expressive. As our system is scalable, it allows users from varied domains and makes it better suited for real-world applications;
→ The scheme is adaptively secure. It achieves resistance to collusion attacks, as the users cannot integrate their attributes to access shared data. The scheme is demonstrated as secure in the standard model.

### 1.4. Organization

The rest of the paper is organized into the following sections: Section 2 compares the traits of prominent schemes studied in the previous section and our scheme. In Section 3, we outline some standard cryptographic definitions and access structure definitions. In Section 4, we propose the system model, the definitions of algorithms, and the security model. The SP-MAACS system construction is illustrated in Section 5. Section 6 discusses the scheme’s security, performance analysis, and implementation results. Section 7 concludes the research work.

## 2. Characteristics Comparison

Table 1 provides a comprehensive comparison of some major characteristics of prominent CP-ABE schemes and SP-MAACS. The comparison involves important features such as multi-authority setting, access policy structure and its expressiveness, privacy preservation, and the security settings of the schemes. The schemes [25,38,39,48] were designed for healthcare systems. From this comparison, we can see that the adaptively secure privacy-preserving schemes [28,38,48] are single-authority schemes. The multi-authority CP-ABE schemes [16,23,25,26] are adaptively secure but do not offer privacy preservation of the access policy. The schemes [35,39] are multi-authority and privacy-preserving schemes, but their security is only evidenced in weaker selective models. As far as our survey goes, the SP-MAACS scheme is the only one that preserves privacy while also offering adaptive security in a multi-authority setting.

## 3. Mathematical Preliminaries

This section introduces the formal definitions and notations of the proposed scheme:

### 3.1. Composite Order Bilinear Groups

The authors defined composite order bilinear groups in [49].

**Definition** **1.** *The order* O*of a bilinear group is defined as*$O=p_{1}p_{2}p_{3}$*, i.e., the product of three different primes (here*$p_{1}$, $p_{2}$*and*$p_{3}).\;$*Let*G*and*$G_{T}$*be cyclic groups of the order* O*. Let the subgroup in*G*with the order*$p_{i}\;$*be denoted as*$\;G_{p_{i}}$*.*$g_{p_{1}},g_{p_{2}}$*and*$\;g_{p_{3}}$*are generators of*$G_{p_{1}}$*,*$\;G_{p_{2}}$*and*$G_{p_{3}}$*, respectively. Let*$e:G\;X\;G\to G_{T}\;$*be the mapping and the following be the required properties:*

*1.*  *Bilinear property:*∀ c,d∈G*and a, b ∈ ZN.*$e\left(c^{a},\;d^{b}\right)=e{\left(c,\;d\right)}^{ab}.$
*2.*  * Property of non-degeneracy:*∃ℊ ∈G, *where*$e\left(g,\;g\right)$*in*$G_{T}\;$*is of the order* O.
*3.*  *Computability:*∃* an algorithm to compute *$e\left(c,d\right)\;\forall c,d\in G\;$*efficiently.*
*4.*  *Orthogonality:*$e\left(g_{p_{1}},g_{p_{2}}\right)\;$*= 1 for any*$g_{p_{1}}$*∈*$\;G_{p_{1}}$*and any*$g_{p_{2}}$*∈*$G_{p_{2}}$.

### 3.2. Access Structure for Privacy Preservation

Here we first define normal access structure.

**Definition** **2.** *Let us name the universe of attributes AU. An access structure*φ *on AU is a collection of non-empty attribute sets, i.e.,*$\;\varphi \subseteq 2^{AU}\backslash \emptyset$*. The collection of attribute sets, which is present in*φ, *is called the authorized set; all the other attributes lie in an unauthorized set. In addition, an access structure is called monotonic if ∀C, D: if C ∈* φ *and C ⊆ D, then D ∈* φ.

Now let us define linear secret sharing scheme (LSSS).

**Definition** **3.** *Let Π be a secret-sharing scheme in which:*

*(1)*  *The generated share for each participant is a vector over*$\;Z_{p}$.
*(2)*  ∃*A matrix W of m rows and n columns, where ∀rows ∈ W, the j^th^ row is marked with the function ρ(j); then it is called a linear scheme. Secret s is randomly chosen such that s ∈*$\;Z_{p}$*and a vector is formed so that*$\;v=\left(s,\;v_{2}\;\ldots \;v_{n}\right)\in Z_{p}^{n}$*. Now let us take λ=*$W\;v^{T}$*such that share*$\lambda_{j\;}$*is for participant ρ(j) so we can write*$\lambda_{j}=W_{j}\cdot \;v$.
*(3)*  *Linear reconstruction property: Let us denote S as an authorized set and take I = {j: ρ(j)∈ S}. For an LSSS scheme, there exists a constant set*$\;{\left(\mu_{j}\in Z_{p}\right)}_{j\in I}$*, used to compute the secret s:*$\underset{j\in I}{\sum }\mu_{j}\lambda_{j}=s$.

*Access Structure for privacy preservation:*

**Definition** **4.** *Let us take an access structure* φ *= (* W *, ρ, Z) for describing an access policy.* W *is a share-generating matrix with the dimensions l by n that is connected with a secret sharing scheme, ρ is a map function and Z =* ${\left(Z_{\rho \left(j\right)}\right)}_{1\leq j\leq l}$ *is a set of corresponding possible values of the respective attribute. Function ρ maps each row of* W *to the name of the attribute present in the access policy. We are keeping Z hidden in our scheme and the share-generating matrix* W  *and function ρ are attached to the ciphertext.*

Disjunctive Normal Form

**Definition** **5.** *In discrete mathematics, a canonical normal form of a Boolean formula can be written as OR of ANDs. It is termed the sum of products (SOP). This normal form is called a disjunctive normal form (DNF). It can be written as* $\;A=A_{1\;}V\;A_{2\;}\;V\;\ldots \;VA_{n\;}\;\left(n\geq 1\right)$ *, where* $A_{1\;},\;A_{2\;},\ldots ,\;A_{n\;}$ *are called sub-formulas and they are all conjunctions of the terms.*

## 4. System Model, Algorithms, and Security Model

This section discusses the system model, various algorithms designed for the scheme, and the security model of the SP-MAACS scheme.

### 4.1. System Model

**Figure 2:**
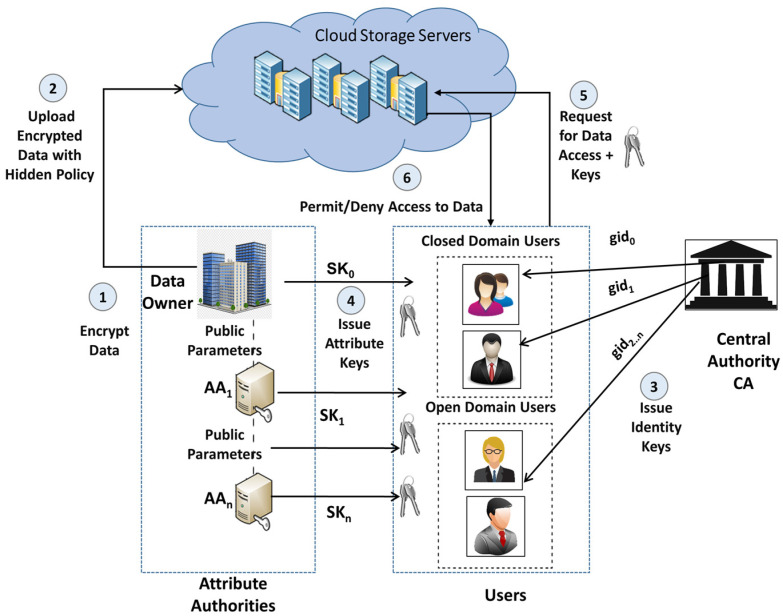
System model for SP-MAACS scheme.

**Figure 3:**
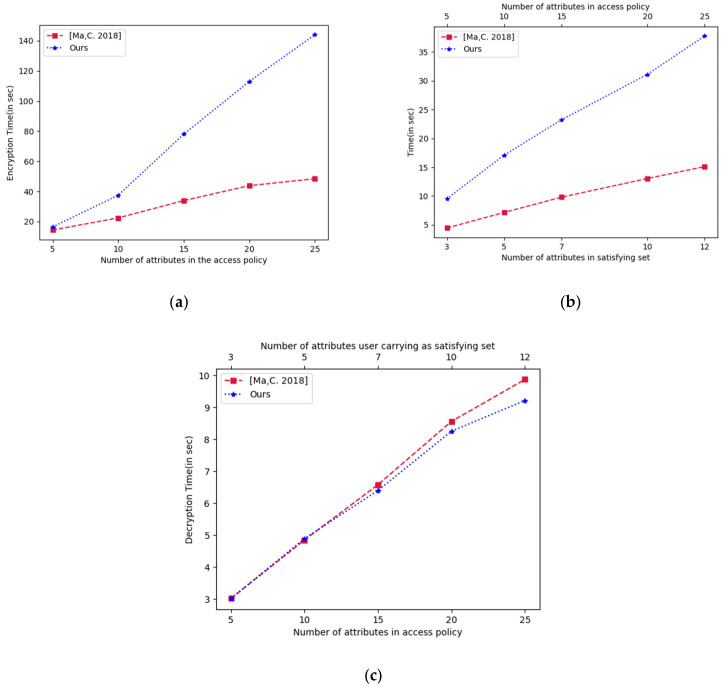
(**a**) Encryption time, (**b**) attribute key generation time, (**c**) decryption time.

Figure 2 shows the major entities in our SP-MAACS system for cloud-based data sharing. The entities are:

(1) ***Data Owner (DO):*** The data owner decides on an access policy and formulates it using the attributes present in the attribute universe. Then they encrypt the data under this policy. These encrypted data are stored on the cloud servers, but the access policy is kept partially hidden (Steps 1 and 2 in Figure 2);
(2) ***Central Authority (CA):*** The responsibilities of a CA can be defined as: (1) Generate global public parameters for the system. (2) Service the user’s request for registration and issue identity keys based on their global identifier (gid) (Step 3 in Figure 2);
(3) ***Attribute Authorities (AAs):*** The responsibilities of an AA can be defined as: (1) Generate public keys for the attributes they manage. Each AA may have the authority to issue any number of attributes, but a single AA is authorized to issue each attribute. (2) Verify the user’s possession of the attribute and issue a secret key for the user’s attributes;
(4) ***Cloud Service Provider (CSP):*** The CSP essentially acts as a resource provider in place of the cloud, replicating that role for the cloud. The data owners use its data storage service and the users send a query for required data to access it. Furthermore, there is an assumption that the CSP is curious about obtaining the knowledge of data, but at the same time, it is honest;
(5) ***User:*** A unique global identity is allotted to every user. They receive a secret key issued for numerous attributes from the responsible AA. The user sends the request for data access to the CSP along with their acquired secret keys, and if the attributes possessed by them are required in satisfying the access policy, they can obtain the data (Steps 5 and 6 in Figure 2).

In the SP-MAACS scheme, attribute authorities in the open domain can be hospitals, clinical research centers, etc. These authorities issue attributes to users such as doctors, nurses, etc. The EHR data owner plays the role of an AA for issuing secret keys to the users in a closed domain, e.g., friends, relatives, etc. (Step 4 in Figure 2).

### 4.2. Algorithms

The SP-MAACS scheme uses the following four algorithms in its construction.

#### 4.2.1. System Initialization

GlobalSetup(λ) → GPP: The algorithm uses the input λ which is also called the security parameter. After the setup executes, global public parameters GPP are generated.

CASetup(GPP) → (MPK, MSK): The central authority(CA) executes the CASetup algorithm. It outputs public key MPK and secret key MSK. All the authorities use MPK for verification purposes.

AASetup(GPP, k, U_k_) → (APK_k_, ASK_k_): Every authority AA_k_ present in the system executes this algorithm, where the inputs are GPP and its attribute domain is called $U_{k}$. No two authorities have a common attribute domain, which means for i ≠ j, $U_{i}\cap U_{j}=\emptyset$. This algorithm produces public key APK_k_ and secret key ASK_k_.

#### 4.2.2. Encryption

Encrypt(K, ψ, GPP, ∪ APK_k_) → (CT): This algorithm takes as input the GPPs, a symmetric key K by which data are encrypted, an access policy structure ψ, and a collection of public keys of applicable authorities. It generates the ciphertext.

#### 4.2.3. User Key Generation

CAKeyGen(GPP,gid) → (CAPK_gid_, CASK_gid_): The user submits their gid as input to this algorithm. Taking GPP as another input, it produces the gid-related identity-key CASK_gid_, which is held by the user. The public key CAPK_gid_ is given to the AAs for generating attribute-related keys.

AAKeyGen($S_{\mathrm{gid},k},$)GPP, MPK, CAPK_gid_, ASK_k_) → (ASK_S,gid,k_): When a user requests k^th^ authority for generating keys for an attribute set $S_{\mathrm{gid},k}$, AA_k_ runs this algorithm with inputs as $S_{\mathrm{gid},k},$ GPP, MPK, CAPK_gid_, and ASK_k_. If CAPK_gid_ is invalid, then it returns ﬩, else it returns corresponding attribute-related keys ASK_S,gid,k_ for attribute set $S_{\mathrm{gid},k}$.

#### 4.2.4. Decryption

Decrypt(CT, GPP, FK_gid_) → (K): The decryption algorithm uses a global public parameter, the ciphertext denoted as CT, and the final secret keys’ set FK_gid_ as the inputs. The decryption is complete when the user’s attributes satisfy the policy.

### 4.3. Security Model

We define the security model for SP-MAACS through a security game between adversary A and challenger C. We assumed that A can corrupt at most K-1 AAs. Let K_c_ denote the index set of corrupted AAs and K_uc_ denote the index set of uncorrupted AAs, where K_uc_ = K\K_c_. The steps of the game are as follows:

**Setup:** C executes the algorithms GlobalSetup, CASetup, and AASetup and transfers the GPP, MPK, and $\cup_{k=1}^{K}APK_{k}$ to the adversary A. Assume the adversary corrupts K_c_ AAs such that K\K_c_ ≠ Φ. The challenger C passes the secret key {ASK_k_ | k∈ K_c_ to A.

**Phase 1:** Adversary A can obtain the secret keys for the AAs who have been corrupted.

**CAKey queries:** For these queries, challenger C responds by CAPK_gid_ and CASK_gid_.

**AAKey queries:** For the attribute set, the adversary submits $S_{\mathrm{gid},k}$ and CAPK_gid_ to C, where k∈ K_uc_. C returns the ${\left\{{\mathrm{ASK}}_{S,\mathrm{gid},k}\right\}}_{k\in K_{uc}}\;\}.$

**Challenge:** If adversary A finds that phase 1 is complete, it sends to C two messages M_0_, M_1_, which are equal in length, and two challenge access structures $\psi_{1}^{*}=\left(W_{1},\;\rho_{1},\;Z_{1}\right)$ and $\psi_{2}^{*}=\left(W_{2},\;\rho_{2},\;Z_{2}\right)$. Here the condition is that $\psi_{1}^{*}\;$ and $\psi_{2}^{*}$ cannot be satisfied by any attribute key query performed in phase 1. A random coin c ∈ {0, 1} is flipped by C; then, it sets $CT_{\psi_{c}}^{*}\leftarrow Encrypt(K_{c},\;\psi_{c},\;GPP,\cup APK_{k})$ and passes this challenged ciphertext to A.

**Phase 2:** As in phase 1, adversary A can again obtain adaptive secret key queries.

**Guess:** Adversary A gives its guess c′ of c as output and wins the game if $c^{\prime }=c$. Probability Pr of being $c^{\prime }=c\;$, i.e., $\left|Pr\left[c^{\prime }=c\right]=\frac{1}{2}\right|$ is called the advantage of adversary A. To compute Pr, A and C choose random bits.

**Definition** **6.** *Our SP-MAACS scheme with a privacy-preserving feature is fully secure since no probabilistic polynomial-time (PPT) adversary has a non-negligible advantage in the above game.*

## 5. Scheme Construction

The following is the detailed construction of SP-MAACS:

### 5.1. System Initialization

The following three algorithms are used for system setup:

**GlobalSetup(λ) → GPP:** The algorithm is called to initialize the system. It uses the input λ and generates GPP (global public parameters). Then, it chooses two bilinear groups G and $G_{T\;}$ of order $O=p_{1}p_{2}p_{3}$ and mapping $\;e:G\;X\;G\to G_{T}$. Let the subgroup in G with the order $p_{i}b$be denoted as $G_{p_{i}}.\;g\;$ is a randomly chosen element from $G_{p_{1}}$. $I_{3\;}$ is a generator of $G_{p_{3}}$. The GPP are made available as =$\;\left(O,\;e,\;g,\;I_{3},\;\Omega_{Sign}\right)$, where $\Omega_{Sign}$= (GenKey,Sign,Verify) is a secure signature scheme and will be used to counter any collusion attempts.

**CASetup(GPP) → (MPK, MSK):** The CA runs the GenKey algorithm of $\Omega_{Sign}$ to obtain signature key MSK and verification key MPK. All the AAs use MPK.

**AASetup(GPP, k, Uk) → (APK_k_, ASK_k_):** The attribute authority AA_k_ runs the algorithm. Let us assume attribute universe AU_k_ consists of n attribute names e.g., (a_1_, a_2_, …, a_n_), and each attribute a_i_ has n_i_ attribute values, e.g., $(a_{i,1},\;a_{i,2}$, …, $a_{i,n_{i}})$. Each AA_k_ chooses a random exponent $t_{k,n_{i}}\in Z_{N}$ and computes $T_{k,n_{i}}=g^{t_{k,n_{i}}}$. It also chooses two random exponents $\alpha_{k},\;\beta_{k}\in Z_{N}$ and computes the public keys of AA_k_ as:

(1) $$PK_{k,1}=e{\left(g,g\right)}^{\alpha_{k}}$$

(2) $$PK_{k,2}=g^{\beta_{k}}$$

Hence, the cumulative public key of AA_k_ is:

(3) $$APK_{k}=\left\{e{\left(g,g\right)}^{\alpha_{k}},g^{\beta_{k}},g^{t_{k,n_{i}}}\forall a_{k,n_{i}}\in U_{k}\right\}$$

And the cumulative secret key of AA_k_ is:

(4) $$ASK_{k}=\left\{\alpha_{k},\beta_{k},t_{k,n_{i}},\;\forall a_{k,n_{i\;}}\in U_{k}\right\}$$

### 5.2. Encryption

When the data owner outsources EHR data, they use a symmetric encryption algorithm and a key K to encrypt the data. Then, they encrypt key K using the following encrypt algorithm with the access policy ψ. Encryption is performed as follows:

**Encrypt(K, ψ, GPP,**$\cup APK_{k}$**)** **→ (CT):** As we mentioned to choose the DNF of a set of sub-policies, let us assume there are q sub-policies, i.e., {ψ_i_}_i=1,2..,q_. For simplicity, let us call each of them W.The sub-policy W is the LSSS matrix. Let us take some rows and columns in the LSSS matrix as land n. The function ρ associates each row $W_{j}$ of W to attribute $\rho \left(j\right)$. To make the policy hidden, a set of attribute values is denoted as $\;Z=(Z_{\rho \left(1\right)},\;\ldots \;Z_{\rho \left(l\right)})$ and is attached to the access policy. Thus, the sub-policy can be expressed by $\left(W,\;\rho ,\;Z\right).\;$For each sub-policy, the following steps are run:

Step 1. The data owner chooses $s\in Z_{p}$ and a random vector

$$v={\left(s,\;v_{2}\;\ldots \;v_{n}\right)}^{T}\in \;Z_{N}^{n}$$

Step 2. For each row of the matrix, the following is calculated:

$$\lambda x=W_{x}\cdot v$$

Step 3. Then, for every $\;x\;\epsilon \;\left[l\right]$, it selects a random exponent $r_{x\;}\epsilon Z_{p}$ and calculates:

(5) $$C=K\cdot \left(\prod_{k\in SAA}e{\left(g,g\right)}^{\alpha_{k}}\right)s=K\cdot e{\left(g,g\right)}^{s}{}^{\underset{k\in SAA}{\sum }\alpha_{k}}$$

(6) $$C_{0}=g^{s}$$

(7) $$C_{1,x}=g^{\sum_{k\in SAA}\beta_{k}\lambda x}\cdot {\left(T_{\rho \left(x\right)}^{Z_{\rho \left(x\right)}}\right)}^{-r_{x}}$$

(8) $$C_{2,x}=g^{r_{x}}$$

Here, SAA is defined as the index set of AA_k_. This set consists of an index of AAs whose attributes are present in the policy.

Step 4. Finally, the data owner sends the ciphertext data to a cloud E(K) = {{ψ_i_}_i=1,2,3,…,q,_ {E_i_}_i=1,2,3,…, q_}, where, $E_{i}=\left\{C,\;C_{0},\;{\left(C_{1,x},\;C_{2,x}\right)}_{x\in \left\{1,\ldots ,l\right\}}\right\}$.

The decryption of the data by the user is possible when their attribute set matches any of the sub-policy $\left(W,\;\rho ,\;Z\right).\;$The matching process is successful when for all $x\in \left\{1,\;\ldots ,\;l\right\},\;a_{\rho \left(x\right)}=Z_{\rho \left(x\right)}$ and constants $\mu_{x}\in Z_{N}$ such that $\;\underset{x\in l}{\sum }\mu_{x}\lambda_{x}=s$.

### 5.3. User Key Generation

Every new user receives a unique gid after he registers himself in the system. For obtaining identity-related keys, he requests CA. The CA runs the CAKeyGen algorithm. After that, the user applies attribute-related keys, and different AAs run the AAKeyGen algorithm for this.

CAKeyGen(GPP,gid) → ($CAPK_{gid}$,${\mathrm{CASK}}_{\mathrm{gid}}$): The CA demands the user’s gid for issuing identity keys. CA randomly chooses $\;r_{gid}\in \;Z_{N}$ and $R_{\mathrm{gid}}\in G_{p_{3}},\;$then sets ${\mathrm{CASK}}_{\mathrm{gid}}=g^{r_{gid}}R_{\mathrm{gid}}$. Then, it uses MSK to compute $\gamma_{gid}=Sign\left(MSK,\;gid∥{\mathrm{CASK}}_{\mathrm{gid}}\right)$. Finally, it sends $CAPK_{gid}=\left(gid,{\mathrm{CASK}}_{\mathrm{gid}},\gamma_{gid}\right)\;$ and ${\mathrm{CASK}}_{\mathrm{gid}}$ to the user.

AAKeyGen($S_{gid,k}GPP,\;MPK,CAPK_{gid},ASK_{k}$) → (ASK_S,gid,k_): To obtain the attribute-related keys issued, a user passes their attribute set $\;S_{\mathrm{gid},k\;}$ to the AA_k_, which belongs to their domain. Then, the AA_k_ uses the MPK to verify whether the ${\;\mathrm{CAPK}}_{\mathrm{gid}}$ is valid. If valid, for issuing keys related to $\;S_{\mathrm{gid},k}$, AA_k_ randomly selects $R_{\mathrm{gid},k}\in G_{p_{3}}$ and computes ${\mathrm{SK}}_{\mathrm{gid},k}=g^{\alpha_{k}}{\mathrm{CASK}}_{\mathrm{gid}}{}^{\beta_{k}}R_{\mathrm{gid},k}=g^{\alpha_{k}}g^{r_{gid}\beta_{k}}{R^{\prime }}_{\mathrm{gid},k}$, where ${R^{\prime }}_{\mathrm{gid},k}=R_{\mathrm{gid}}{}^{\beta_{k}}R_{\mathrm{gid},k}$, else it aborts.

For each attribute $\;i\;\in S_{\mathrm{gid},k}$, it randomly selects ${\;R}_{\mathrm{gid},k,i}\in G_{p_{3}}$ and computes ${\mathrm{SK}}_{\mathrm{gid},k,i}={\mathrm{CASK}}_{\mathrm{gid}}{}^{t_{k,ni}}R_{\mathrm{gid},k,i}=T_{k,ni}{}^{r_{gid}}{R^{\prime }}_{\mathrm{gid},k,i}$, where ${R^{\prime }}_{\mathrm{gid},k,i}=R_{\mathrm{gid}}{}^{t_{k,ni}}R_{\mathrm{gid},k,i}$. Finally, ASK_S,gid,k_ = $\left({\mathrm{SK}}_{\mathrm{gid},k},{\left\{{\mathrm{SK}}_{\mathrm{gid},k,i}\right\}}_{i\;\in S_{\mathrm{gid},k}}\right)$ is given to the user.

Therefore, the final set of user keys FK_gid_ contains:

(9) $$K1=g^{r_{gid}}R_{\mathrm{gid}}$$

(10) $$K2=\text{\{}{\mathrm{SK}}_{\mathrm{gid},k}\}$$

(11) $$K_{3}=\text{\{}\left({\mathrm{SK}}_{\mathrm{gid},k,i}{}_{i\;\in S_{\mathrm{gid},k}}\right)\}$$

### 5.4. Decryption

When a user submits their data access request to the cloud server along with their secret keys, the decrypt algorithm is executed as follows:

Decrypt(CT, GPP, FK_gid_)→(K): When the set of attribute keys of the user (S_gid_) matches any of the conjunction or sub-policy $\left(W,\;\rho ,\;Z\right)$, then the symmetric key K can be retrieved by the following steps:

Step 1: Compute $CK=\underset{i\in SAA}{\prod }K_{2,i},$ and choose constants $\mu_{x}\in Z_{N}$, such that $\underset{\rho \left(x\right)\in S_{\mathrm{gid}}}{\sum }\mu_{x}W_{x}=\left(1,0,\ldots ,0\right)$. Then compute

(12) $$e\left(\;CK,\;C_{0}\right)=e\left(\prod_{k=1}^{l}g^{\alpha_{k}}g^{r_{gid}\beta_{k}}{\;R^{\prime }}_{\mathrm{gid},k},\;g^{s}\right)=e{\left(g,g\right)}^{s\overset{l}{\underset{k=1}{\sum }}\alpha_{k}}e{\left(g,g\right)}^{r_{gid}s\overset{l}{\underset{k=1}{\sum }}\beta_{k}}$$

Step 2: For all attribute-related keys for which $\rho \left(x\right)\in S_{\mathrm{gid}}$, compute

(13) $$\prod_{\rho \left(x\right)\in S_{\mathrm{gid}}}\;{\left[e\left(C_{1,x},K_{1}\right)e\left(C_{2,x},K_{3,i}\right)\right]}^{\mu_{x}}=\;\prod_{\rho \left(x\right)\in S_{\mathrm{gid}}}{\left[\;e\left(g^{\sum_{k\in SAA}\beta_{k}\lambda x}\cdot {\left(g^{t_{\rho \left(x\right)}Z_{\rho \left(x\right)}}\right)}^{-r_{x}},\;g^{r_{gid}}R_{\mathrm{gid}}\right)\;e\left(g^{r_{x}},\;T_{\rho \left(x\right)}^{Z_{\rho \left(x\right)}}{}^{r_{gid}}{\;R^{\prime }}_{\mathrm{gid},k,i}\right)\right]}^{\mu_{x}}\;=\prod_{\rho \left(x\right)\in S_{\mathrm{gid}}}{\left[\;e\left(g^{\sum_{k\in SAA}\beta_{k}\lambda x},g^{r_{gid}}\right)\right]}^{\mu_{x}}=e{\left(g,g\right)}^{r_{gid}s\sum_{k\epsilon SAA}\beta_{k}}$$

Step 3: After dividing the result of step 1 by step 2, we obtain $e{\left(g,g\right)}^{s\overset{l}{\underset{k=1}{\sum }}\alpha_{k}}$.

Step 4:

(14) $$C/\;e{\left(g,g\right)}^{s\overset{l}{\underset{k=1}{\sum }}\alpha_{k}}=K\cdot \;e{\left(g,g\right)}^{s}{}^{\underset{k\in SAA}{\sum }\alpha_{k}}/\;e{\left(g,g\right)}^{s\overset{l}{\underset{k=1}{\sum }}\alpha_{k}}=K$$

The user can recover the data by using the symmetric key K.

## 6. Security and Performance Analysis

### 6.1. Security Analysis

The following complexity assumptions serve as the foundation for our security proofs:

Subgroup Decision Problem for Three Primes [38,50]

Three assumptions are contained in this SDP assumption. Here Pr denotes the probability function.

**Assumption** **1:** *Let us take a group generator* $\overset{\text{-}}{g}$ *and consider the distribution:* G *=(* $O=p_{1}p_{2}p_{3},G,G_{T},e$ *)* $\overset{R}{\leftarrow }\;\overset{\text{-}}{g},\;g\overset{R}{\leftarrow }G_{p_{1}},X_{3}\overset{R}{\leftarrow }G_{p_{3}},$ *D = (* $G,g,X_{3}$ *),* $T_{1}\overset{R}{\leftarrow }G_{p_{1}P_{2}},T_{2}\overset{R}{\leftarrow }G_{p_{1}}.\;$ *The advantage of an algorithm* A *in breaking the mentioned assumption is:*

(15) $$SDP\_Adv1_{G,A}\left(\lambda \right)=|Pr\left[\;A\left(D,T_{1}\right)=1]-\mathrm{Pr}[\;A\left(D,T_{2}\right)=1\right]$$

**Definition** **7.** *If for any polynomial time (PT) algorithm* A *,* $\;SDP\_Adv1_{G,A}$ *(* λ *) is a negligible function of* λ *, then* $\overset{\text{-}}{g}$ *is said to satisfy the above-mentioned assumption 1.*

**Assumption** **2:** *Let us take a group generator* $\overset{\text{-}}{g}$ *and consider the distribution:* G *= (* $O=p_{1}p_{2}p_{3},G,G_{T},e$ *)* $\overset{R}{\leftarrow }\;\overset{\text{-}}{g},g,X_{1}\overset{R}{\leftarrow }G_{p_{1}},X_{2},Y_{2}\overset{R}{\leftarrow }G_{p_{2}},X_{3},Y_{3}\overset{R}{\leftarrow }G_{p_{3}},$ *D = (* $G,g,X_{1}X_{2},X_{3},Y_{2}Y_{3}$ *),* $T_{1}\overset{R}{\leftarrow }G,T_{2}\overset{R}{\leftarrow }G_{p_{2}P_{3}}$ *. The advantage of an algorithm* A *in breaking the mentioned assumption is:*

(16) $$SDP\_Adv2_{G,A}\left(\lambda \right)=\left|Pr\left[A\left(D,T_{1}\right)=1]-Pr[A\left(D,T_{2}\right)=1\right]\right|$$

**Definition** **8.** *If for any polynomial-time (PT) algorithm* $A,SDP\_Adv2_{G,A}$ *(* λ *) is a negligible function of* λ *, then* $\overset{\text{-}}{g}$ *is said to satisfy the above-mentioned assumption 2.*

**Assumption** **3:** *Let us take a group generator* $\overset{\text{-}}{g}$ *and consider the distribution:* G *=(* $O=p_{1}p_{2}p_{3},G,G_{T},e$ *)* $\overset{R}{\leftarrow }G,\alpha ,s\overset{R}{\leftarrow }{\mathbb{Z}}_{N},g\overset{R}{\leftarrow }G_{p_{1}},X_{2},Y_{2},Z_{2}\overset{R}{\leftarrow }G_{p_{2}},X_{3}\overset{R}{\leftarrow }G_{p_{3}},$ *D=(* $G,g,g^{\alpha }X_{2},X_{3},g^{s}Y_{2},Z_{2}$ *),* $T_{1}=e{\left(g,g\right)}^{\alpha }s,T_{2}\overset{R}{\leftarrow }G_{T}$ *. The advantage of an algorithm* A *in breaking the mentioned assumption is:*

(17) $$SDP\_Adv3_{G,A}\left(\lambda \right)=\left|Pr\left[A\left(D,T_{1}\right)=1]-Pr[A\left(D,T_{2}\right)=1\right]\right|$$

**Definition** **9.** *If for any polynomial-time (PT) algorithm*$A,\;SDP\_Adv3_{G,A}$*(*λ*) is a negligible function of*λ, *then*$\overset{\text{-}}{g}$*is said to satisfy the above-mentioned Assumption 3*.

**Theorem** **1.** *If the above three assumptions are true, then the proposed SP-MAACS scheme is fully secure according to the model presented in* Section 4.3 *—Definition 6.*

**Proof.**  We will use two terms here: ciphertext in semi-functional form (SF-CT) and key in semi-functional form (SF-Key). The terms are used in proof [16] and are not used in the construction of the scheme. We chose a random exponent $z_{k,i}\in Z_{N}$ for each attribute i $\in U_{k}$. □

Semi-functional ciphertext. To make an SF-CT, perform the following:

Let $g_{2}$ be a generator of$\;G_{p_{2}}$, $d\overset{R}{\leftarrow }Z_{N}$. ∀row$\;x\epsilon \left[l\right]$, let us randomly select ξx$\;\in Z_{N}$. In addition, choose a random vector$\vec{\;y}\;\in Z_{N}^{n}$. Then, set

$$C_{0}=g^{s}\cdot g_{2}{}^{d}$$

For each row$\;x\;\epsilon \;\left[l\right]$,

$$C_{1,x}=g^{\sum_{k\in SAA}\beta_{k}\lambda x}\cdot {\left(T_{\rho \left(x\right)}^{Z_{\rho \left(x\right)}}\right)}^{-r_{x}}\cdot \;g_{2}{}^{W_{x}.\vec{y}+\xi xz_{\rho \left(x\right)}}$$

$$C_{2,x}=g^{r_{x}}\cdot g_{2}{}^{-\xi x}$$

Semi-functional key: There can be two types:

Type 1 SF-Key: Choose random exponents r, δ_k_$\in Z_{N}\;$and set:

$${\mathrm{CASK}}_{\mathrm{gid}}=g^{r_{gid}}R_{\mathrm{gid}}\cdot g_{2}{}^{r}$$

$$CAPK_{gid}=\left(gid,{\mathrm{CASK}}_{\mathrm{gid}},\gamma_{gid}\right)$$

$${\mathrm{SK}}_{\mathrm{gid},k}=g^{\alpha_{k}}g^{r_{gid}\beta_{k}}{R^{\prime }}_{\mathrm{gid},k}\cdot g_{2}{}^{\delta_{k}}$$

$${\mathrm{SK}}_{\mathrm{gid},k,i}=T_{k,ni}{}^{r_{gid}}{R^{\prime }}_{\mathrm{gid},k,i}\cdot g_{2}{}^{rz_{k,i}}$$

Type 2 SF-Key:

$${\mathrm{CASK}}_{\mathrm{gid}}=g^{r_{gid}}R_{\mathrm{gid}}$$

$$CAPK_{gid}=\left(gid,{\mathrm{CASK}}_{\mathrm{gid}},\gamma_{gid}\right)$$

$${\mathrm{SK}}_{\mathrm{gid},k}=g^{\alpha_{k}}g^{r_{gid}\beta_{k}}{R^{\prime }}_{\mathrm{gid},k}\cdot g_{2}{}^{\delta_{k}}$$

$${\mathrm{SK}}_{\mathrm{gid},k,i}=T_{k,ni}{}^{r_{gid}}{R^{\prime }}_{\mathrm{gid},k,i}$$

If we use a regular key to decipher an SF-CT or an SF-Key to decipher a regular ciphertext, we can correctly calculate $\left(\underset{k\in SAA}{\prod }e{\left(g,g\right)}^{\alpha_{k}}{}^{s}\right)$. However, if we try to use an SF-Key to decipher a semi-functional CT, it will give us an extra thing:$\;e{\left(g_{2},g_{2}\right)}^{c}{}^{\underset{k\in SAA}{\sum }\delta_{k}-ry_{1}},\;$where y_1_ is the first coordinate of the vector $\vec{y}$.

The adaptive security of the scheme from three assumptions (Assumption no. 1, 2, 3), can be confirmed using a sequence of games shown in the appendix of [38].

### 6.2. Performance Analysis

As shown in Table 1, our scheme is compared with prominent multi-authority schemes, single-authority privacy-preserving schemes, and some privacy-preserving multi-authority schemes. The salient features of our scheme are also highlighted in Section 1 and Section 2. Table 2 shows the comparison of storage overhead and encryption and decryption computation costs. Table 3 presents a summary of the notations that are used in the comparison.

From the above numerical performance analysis, it can be observed that the key generation time of our scheme and schemes [23,39] are comparable, while other schemes have shorter key generation times. The reason for this is that either they are single-authority or not provide privacy preservation features. The numerical decryption time of our scheme is less than that of the others, as the number of exponentiation operations performed is less.

### 6.3. Implementation Result

Through the characteristic comparison presented in Table 1, our scheme SP-MAACS has been shown to be better than other schemes in terms of features attained. We implemented our scheme and the fully secure decentralized CP-ABE scheme [26] and assessed the performance. The authors of fully secure decentralized CP-ABE presented two constructions [26] and demonstrated them to be secure under the standard model. Their first construction used a composite-order bilinear group and was confirmed to be fully secure by taking static assumptions. The scheme applies to any monotone access structure.

#### 6.3.1. Implementation Environment

The SP-MAACS scheme and the fully secure decentralized CP-ABE scheme [26] were implemented using the well-developed and robust JPBC library [51]. We used the Eclipse IDE to implement the simulation code and the code was written in Java. The tests were conducted on a laptop running Windows 10 (64-bit) and equipped with a 2.50 GHz Intel (R) i5-3210M processor and 4 gigabytes of RAM.

#### 6.3.2. Implementation Setup

ABE schemes can be implemented using pairing-based cryptography. The Java Pairing Based Cryptography JPBC library [51] is extensively used in developing cryptographic solutions. While configuring JPBC, the following .jar files are included: jpbc-pbc-v2.0.0-m.jar, jpbc-plaf-v2.0.0-m.jar, jpbc-api-v2.0.0-m.jar, jpbc-crypto-v2.0.0-m.jar, and bcprov-jdk16-1.46.jar. JPBC includes various packages such as api, util, pairing, field, etc. Some interfaces for pairing functions, finite fields, and elliptic curves are provided by package api. Package util offers support for mathematical operations and other functions. Package pairing and field are the concrete realizations of the interfaces offered by package api. JPBC supports a variety of elliptic curve types. Type A, Type A1, Type D, Type E, Type F, and Type G are included. This experiment was conducted on the group of elliptic curves of Type A1. Their order is the product of three primes of length 517 bits. In the global setup method, while performing setup for the central authority, the Boneh–Lynn–Shacham signature scheme, called the BLS signature, was implemented. A BLS signature enables a user to validate the authenticity of a signer. Signatures are created as elements in elliptic curve groups and verified using a pairing function. The Junit testing framework was used for testing the implemented classes.

#### 6.3.3. System Setup

Exponentiations and pairing operations in the above algorithms for the encryption and decryption process account for a large portion of the computational overhead of CP-ABE systems; thus, we analyzed the encryption and decryption cost of our scheme. For the analysis purpose, we assumed that in our system, five attribute authorities are responsible for attribute management. In addition, it was assumed that each authority manages five attributes. Each attribute may have any one of the possible attribute values. We also implemented a secure signature scheme, which was used by CA to sign the user key, and AA use verified the algorithm to perform the verification of the key.

#### 6.3.4. Result Analysis

Figure 3 illustrates the computation time of the encryption, key generation, and decryption algorithms. Each algorithm’s experimental outcome was the mean of 10 independent runs. Figure 3a presents a graph between the encryption time and the attribute count in the access control policy. We took several attributes on the X-axis in the range of 5 attributes to 25 attributes. We have already mentioned that our scheme considers the DNF access structure and the scheme [26] uses a traditional AND-OR access structure. Typically, when a data owner decides on an access policy, they start to build it by combining many possibilities using the “OR” gate. This standard method of specifying access policy corresponds to the DNF structure used by our approach. The time taken for encryption in our scheme is higher because we perform encryption for every access sub-structure clause. This, in turn, increases the number of ciphertext components. It can be observed that the encryption activity is less frequent. Due to the vast computational power and storage capacities of the cloud, increased encryption time and storage needs are acceptable.

Figure 3b shows the key generation time versus attribute count in the satisfying set. In SP-MAACS, the key generation time is higher, as the user secret key contains the identity and attribute-value-related key components in addition to the authority-related keys. We use identity and attribute-value-related keys to provide collision resistance and privacy preservation features. The central authority CA of the system registers the user and issues them identity-related keys, and are the keys are issued to the user by the CA (shown in the construction of the scheme). The user may have multiple values for their attributes. The attribute authority AA issues both authority-related and attribute-value-specific keys to the user. Scheme [26], on the other hand, only considers keys provided by the attribute authority. Therefore, we compromise key generation time to deliver more functionality. Figure 3c shows a graph between the decryption times and attribute count. In Figure 3c, attributes present in policy and attribute count in the satisfying sets are shown on the dual X-axis. While performing decryption, the user can determine the smallest satisfying set out of the policy defined by the owner of the data. In general, we can assume that on average 50% of the attributes make a satisfying subset out of the total attributes present in the policy. For example, if an access control policy contains 20 attributes in it, then in general the AND-OR combination requires that the user should carry on average 10 attributes for satisfying it or the policy require only 10 attributes to fulfill it. The DNF clause, which matches the attribute and its value in the user-supplied keys, is only utilized during the decryption process. Since there is no requirement for attribute matching with the whole access structure, our scheme takes less decryption time and improves decryption efficiency in comparison to the scheme [26]. This improvement in decryption time satisfies the major requirement of healthcare data sharing, where a doctor wants to access a patient’s EHR data quickly in a life-critical situation.

In summary, our scheme simultaneously provides features of multi-authority, privacy preservation, efficient decryption, and adaptive security. The increase in storage cost and encryption time is affordable, as cloud storage is available at a very nominal cost and encryption activity is performed less frequently. The users may ask for the download and decryption of the ciphertext randomly as per their requirement, so it is a frequent process. Thus, reducing the decryption cost is beneficial.

## 7. Conclusions

Today, the cloud is the most obvious data-sharing platform for the healthcare sector, and ABE schemes can be used to provide access control on outsourced EHR data in encrypted form. In order to share data on cloud storage servers, this article suggests the SP-MAACS scheme, a completely secure and privacy-preserving multi-authority access control system for cloud-based healthcare data sharing. Data owners may now freely share their data with all users in both open and closed domains. This makes the system scalable and adaptable. The partially hidden access policy protects user as well as data owner privacy. Our implementation results demonstrate that the scheme achieves an improvement in decryption cost despite the scheme being privacy-preserving and providing adaptive security under the standard model. The efficiency of decryption can be further increased by outsourcing the decryption to proxy servers in the future. Healthcare data management and privacy protection are currently one of the most active blockchain research areas. Combining the proposed control scheme with blockchain technology could improve security, privacy, and audibility.

## Figures and Tables

**Table 1:** Prominent ABE schemes vs. SP-MAACS scheme.

Scheme	CP/KP	Multi- Authority	Privacy-Aware	Expressiveness	Security	Group Order	Security Model
**Single-Authority Privacy Preserving Schemes with Adaptive Security in Standard Model**							
[28]	CP	×	√ (Partially hidden policy)	LSSS	Adaptive	Composite	Standard
[38]	CP	×	√ (Partially hidden policy)	LSSS	Adaptive	Composite	Standard
[48]	CP	×	√ (Partially hidden policy)	LSSS	Adaptive	Composite	Standard
**Multi-Authority Schemes**							
[16]	CP	√	×	LSSS	Adaptive	Composite	Random oracle
[23]	CP	√	×	LSSS	Adaptive	Composite	Standard
[26]	CP	√	×	LSSS	Adaptive	Composite	Standard
[25]	KP	√	×	LSSS	Adaptive	Composite	Standard
**Multi-Authority Privacy Preserving Schemes**							
[35]	CP	√	√ (Fully hidden policy)	LSSS	Selective	Prime	Random oracle
[39]	CP	√	√ (Partially hidden policy)	LSSS	Selective	Prime	Standard
**Multi-Authority Privacy Preserving (Partially Hidden policy) Scheme with Adaptive Security in Standard Model**							
**Ours**	CP	√	√ (Partially hidden policy)	LSSS	Adaptive	Composite	Standard

**Table 2:** Storage and computation cost comparison.

Scheme	Storage Overhead			Computation Cost	
	Public Key	User’s Secret Key	Ciphertext	Encryption	Decryption
[28]	$\left|G_{T}\right|+\left(n+4\right)\left|G\right|$	$\left(\left|A_{U}\right|+2\right)\left|G\right|$	$\left(4\left|{\;A}_{C}\right|+2\right)\left|G\right|+2\left|G_{T}\right|$	$\left(7\;\left|{\;A}_{C}\right|+4\;\right)\;E$	$\left(2\left|I\right|+4\right)P+\left(4\left|I\right|\right)E$
[38]	$\left|G_{T}\right|+4\left|G\right|$	$\left(\left|A_{U}\right|+2\right)\left|G\right|$	$\left(3\left|{\;A}_{C}\right|+2\right)\left|G\right|+2\left|G_{T}\right|$	$\left(7\;\left|{\;A}_{C}\right|+4\;\right)\;E$	$\left(2\left|I\right|+3\right)P+\left(3\left|I\right|\right)E$
[16]	$n\left|G_{T}\right|+n\left|G\right|$	$\left(\left|A_{U}\right|\right)\left|G\right|$	$\left(2\left|{\;A}_{C}\right|\right)\left|G\right|+\left(\left|{\;A}_{C}\right|+1\right)\left|G_{T}\right|$	$\left(5\;\left|{\;A}_{C}\right|+1\;\right)\;E$	$\left(2\left|I\right|\right)P+\left(\left|I\right|\right)E$
[23]	$n_{a}\left|G_{T}\right|+\left(n+n_{a}+1\right)\;\left|G\right|$	$\left(n_{a}+\left|A_{U}\right|+2\right)\left|G\right|$	(2$\left|A_{C}\right|+1)\left|G\right|$ +$\left|G_{T}\right|$	$\left(\;3\left|A_{C}\right|+2\right)E$	$\left(2\left|I\right|+1\right)P+\left(\left|I\right|+1\right)E$
[26]	$n\left|G_{T}\right|+\left(n+1\right)\left|G\right|$	$\left(\left|A_{U}\right|+1\right)\left|G\right|$	$\left(2\left|{\;A}_{C}\right|\right)\left|G\right|+\left(\left|{\;A}_{C}\right|+1\right)\left|G_{T}\right|$	$\left(5\;\left|{\;A}_{C}\right|+1\;\right)\;E$	$\left(2\left|I\right|\right)P+\left(3\left|I\right|\right)E$
[35]	$n\left|G_{T}\right|+n\left|G\right|$	$\left(2\left|A_{U}\right|\right)\left|G\right|$	$\left(2\left|{\;A}_{C}\right|+1\right)\left|G\right|+\left(\left|{\;A}_{C}\right|+1\right)\left|G_{T}\right|$	$\left(5\;\left|{\;A}_{C}\right|+1\;\right)\;E$	$\left(2\left|I\right|+1\right)P+\left|I\right|E$
[39]	$n_{a\;}\left|G_{T}\right|+\left(n+{\;n}_{v}\right)\left|G\right|$	$\left(n_{a}+\left|A_{U}\right|\right)\left|G\right|$	(2$\left|A_{C}\right|+1)\left|G\right|+\left|G_{T}\right|$	$\left(5\left|{\;A}_{C}\right|+1\right)E$	$\left(3\left|I\right|\right)P+\left|I\right|E$
**[Ours]**	$n_{a\;}\left|G_{T}\right|+\left(n+{\;n}_{v}+1\right)\left|G\right|$	$(n_{a}+\left|A_{U}\right|+2)\left|G\right|$	(2$\left|A_{C}\right|+1)\left|G\right|$ +$\left|G_{T}\right|$	$\left(\;3\left|A_{C}\right|+2\right)E$	$\left(2\left|I\right|+1\right)P+\left|I\right|\;E$

**Table 3:** Summary of notations.

Notation	Description
** $\left|G\right|$ **	No. of bits needed to represent an element in group G
** $\left|G_{T}\right|$ **	No. of bits needed to represent an element in group$\;G_{T}$
** $\left|I\right|$ **	No. of attributes of satisfying set
** n **	The size of universe of attributes
** $A_{C}$ **	Attribute set used in encryption
** $A_{U}$ **	User’s attribute set
** E **	One exponential operation
** P **	One pairing operation
** $n_{a}$ **	Count of AAs in the system
** $n_{v}$ **	Number of values all the attributes in the system may have (an attribute may have multiple values)

## Data Availability

Available on request.
